# The Effectiveness of Community Sports Provision on Social Inclusion and Public Health in Rural China

**DOI:** 10.3390/ijerph17020597

**Published:** 2020-01-17

**Authors:** Qiu Chen, Tianbiao Liu

**Affiliations:** 1 China Center for Agricultural Policy (CCAP), School of Agricultural Sciences, Peking University, No. 5 Yiheyuan Road, Beijing 100871, China; qchen.ccap@pku.edu.cn; 2 National School of Development, Peking University, No. 5 Yiheyuan Road, Beijing 100871, China; 3 College of Physical Education and Sports, Beijing Normal University, No. 19 Xinjiekouwai Street, Beijing 100875, China

**Keywords:** community sports provision, public sports facilities, organized sports activities, social inclusion, public health

## Abstract

It is well known that increasing participation in physical activities is not only positive for individual health promotion, but also beneficial for community-level public health by enhancing the individual’s social well-being by facilitating social inclusion. Although the provision of community sports affects participation in physical activities, the magnitude and direction of this effect are still not clear. Under this circumstance, this paper examined the effects of community sports provision on social inclusion and public health using the micro-level data from a household survey conducted in eight provinces of rural China. For the purpose of this paper, the degree of social inclusion was proxied by participation in community sports, while public health was measured by the probability of getting ill for members of each household. The empirical results show that community sports provision was partially effective in promoting inclusion and health in rural areas. Specifically, constructing public sports facilities significantly increases participation in community sports and decreases the risk of getting ill. In contrast, organizing public sports activities increases the opportunity for households to play sports. Nonetheless, it does not promote public health. Equally important is that economic growth (reflected in the increase in income level) may enhance public health through advancing medical technologies and improving sanitary conditions instead of encouraging participation in community sports.

## 1. Introduction

Health has been found to be both human capital itself and an input to produce other forms of human capital [1]. Thus, enhancing health has always been an important strategy for building human capital to assist sustainable rural development in China. Over the last three decades, China has experienced rapid and massive urbanization. Hundreds of millions of rural workers have migrated to cities, resulting in a series of social problems, such as increasing unemployment, rising crime rate, and widening urban-rural gap [2]. Moreover, due to the fact that compared to the labor force remaining in rural areas, migrant workers are younger and more educated with relatively higher human capital. Rural-urban migration also caused brain drain in rural regions, decreased agricultural labor productivity, and inflated price of agricultural products [3]. Hence, encouraging labor migrants to return back home is of strategic importance for social and economic development of rural China. In recent years, the Chinese government has had a number of initiatives and programs to facilitate the settlement of returned migrant workers in rural local communities. Specifically, as a form of social welfare policy intervention, provision of inclusive sports is an effective instrument to increase sport participation, aiming at not only promoting physical and mental health, but also improving community cohesion [4]. It may also play a crucial role in fostering a sense of community belonging for recently returned migrant workers and strengthen communication among all members [5]. In the current context, sport is considered to include formal governing body-related activities and informal activities performed for recreational purposes using public sports facilities, such as square dancing, racquet and ball sports, and running/jogging, while sport participation refers to mass participation in team or individual sports that can take place in open public spaces or with public sport infrastructure. As there has been little attention given to the supply side of community sport provision on participation or on local populations’ health, it needs to be better understood whether the current community sports provision can increase sport participation to enhance social inclusion and public health within local communities in rural China.

The health benefits of sport participation are well known. Numerous recent studies from a wide range of disciplines have shown that the increased participation in physical activities is positive for individual health promotion [6,7,8,9]. At present, a physically inactive lifestyle has been recognized as a major contributor to the obesity epidemic [10], as well as to the burden of chronic diseases including diabetes and cardiovascular disease [11]. Moreover, sport participation also has a positive impact on the well-being of individuals [4]. Physically active participants usually experience less depression, a stronger sense of coherence, and less perceived stress in comparison to those who exercise less frequently. They are more satisfied with their lives and less likely to suffer from mental health problems [12]. On the other hand, the role of sports participation in supporting community-based public health has received considerable attention in recent research. Within a community, sport participation is one of the important sources through which social interactions could be achieved that might increase the ability of individuals to engage meaningfully in local society, thereby improving the social capital of the whole population and the social inclusion [13].

The concept of social inclusion emerged and basically developed as a response to the issue of social exclusion in Europe [14,15]. As proposed by Berman and Phillips [16], social inclusion is an expression of social quality. It aims at achieving a basic level of inclusion with help of supportive infrastructures, labor conditions, and collective goods in such a way that those mechanisms causing exclusion (i.e., inadequate social participation, lack of social integration, and lack of power) will be prevented or minimized [12,16]. In the context of sport and social inclusion, several studies have been conducted to examine the benefits of sport to the enhancement of social inclusion. To fit sport into the discussion of social inclusion, Ponic and Frisby [17] conceptualized social inclusion as an ongoing relational process whereby people and organizations actively and collaboratively co-create spaces and structures that enable community members to make decisions about how and when to participate in physical activity and sport. They also provided the participatory dimension of the social inclusion framework, which refers to whether participants have the opportunity to choose to be involved in their communities and the variety of ways that they can do so [17]. Along with this, there is a growing body of sport management literature on social inclusion [18,19,20,21]. Among them, Liu [14], and Hodgkinson and Hughes [22] focused on the inclusion of disadvantaged groups in public leisure activities in the UK, while Pitts and Shapiro [23] explored the inclusion of people with disabilities in the sport business industry and sport management of the United States. Frisby [24] and Forde et al. [25] investigated the role of sport in in the settlement of immigrants in Canada, while Shaw [26] examined the potential of anti-homophobia policy to develop social inclusion through community sport in New Zealand. The findings of these studies highlighted the access and the utilization of public sports facilities and the participation in community sport as important performance indicators, measuring the achievement of social inclusion through sport. They also suggested that sport has been regarded as an effective tool to increase social inclusion in the developed world. However, little attention has been paid to this issue in developing countries. As the large rich-poor gap and unbalanced development of the regional economy led to a series of social problems, including health disparities and social inequality [27], how to provide equal opportunities for all citizens irrespective of their socioeconomic status is at the heart of the social equity concern for the developing countries. Particularly in the field of sport research, it is of great significance to reveal the role of sports provision in propelling social inclusion. 

The structure of the rest of this study is the following: the development status of public sports services will be presented in Section 2. Section 3 describes the dataset used for analysis in this study. The empirical strategy, including the model specification and variable selection, will be given in Section 4. Section 5 reports the estimation results of the empirical models, and Section 6 will conclude and discuss the main findings of this study. 

## 2. Community Sports Provision in Rural China 

The provision of community sports in rural China is a kind of government-led institutional arrangement (Unlike the concept of “community sports” provided in the literature focusing on many developed countries, the “community sports” are usually termed as “public sports services” in the majority of studies in China. It is defined as sports facilities, activities, and related education or trainings, programs provided by the government as public goods, aiming at increasing public health and social well-being in rural China [23,28,29,30]. Different from the “community sports” in western countries, the provision of community sports in rural China is not to focus on a specific target group’s need. Instead, it is an approach to increase participation of the whole population to alleviate health disparities and build social capital within a specific community (village)). As defined by Peng and Pang [31], community sports provision, in the context of rural China, is regarded as a community-level intervention tool to promote health and well-being. It is part of the public goods and services supply. It consists of sports facilities and organized physical activities provided by the government, sports information services (including sports news, shows and special-event reports, etc.) supplied by community advocacy, as well as sports initiatives and programs set up through collaborations between various local actors, such as public sport organizations and local sports services within rural communities [23]. It is characterized by: (1) being a highly centralized and unified planning system, (2) the dominant position of government investment, and (3) the use of single organizational format and supply pattern [23,29]. The main model of community sports in rural China is “participation,” with sport being a goal for the whole population in a specific village [28,29]. The Chinese government expects to achieve urban-rural integration and narrow the urban-rural gap by simultaneously reducing health inequalities and increasing social connections. Thus, considering that sports participation can offer a range of physical and psychosocial benefits, the provision of community sports could be regarded as a means of increasing the opportunities for social interactions. In other words, increasing sports participation to integrate the newly settled members into the community and to build human and social capital for facilitating inclusion and development is the main purpose of community sports provision in rural China [29].

Since the Chinese government has realized that sports participation is not only an important instrument in social policy with the capacity to enhance public health, but also a mediator for promoting social inclusion, it started to implement regulations and nationwide fitness programs to support the provision of community sports. The Sports Law of the People’s Republic of China was enacted by the standing committee of the National People’s Congress and the National Fitness Program Outline was launched by the State Council in June and August of 1995, respectively. These regulatory documents suggest strengthening people’s physique and facilitating the formation of a healthy lifestyle by encouraging public participation in physical activities. In August 2009, the first national specialized policy, Regulations on National Fitness Program, was promulgated by the State Council based on the Plan for National Fitness Program (2011–2015), which was issued in the spring of 2011. Both of these two policies aimed to achieve the goals that the per capita area of sports field is more than 1.5 square meters in rural areas and over 50% of villages have public sports facilities and fitness stations by 2015. They also provide legal support for local governments to design public sports areas in rural communities and villages. Although the provision of community sports has been regarded as an important indicator for measuring the level of regional socioeconomic development, the public sports participation rates are still low in rural areas [30]. Under this circumstance, the State of General Administration of Sports (SGAS) had successively implemented the “Farmers Fitness Project,” “Township Fitness Project” and “Xue-Tan Project” (“Xue-Tan” is a Chinese word that means “provide timely help”) to vigorously promote the construction of public sports facilities in rural areas, as well as to call for efforts and actions from provincial governments since 2006. In 2016, the SGAS and the State Council separately issued the 13th Five-Year (2016–2020) National Plan for Sports Development and the Plan for National Fitness Program (2016–2020) and extended the goals to more than 1.8 square meters sports field per capita and 100% coverage rate of fitness facilities in rural areas, to be achieved by 2020. In addition to proposing more specific measures, such as reforming wasteland into sports fields and organizing sports competitions, these plans also suggest social capital to invest in the construction of rural public sports facilities.

Currently in rural China, basketball courts and table tennis tables still dominate in public sports facility provision and a high proportion of the budget goes to them. According to the bulletin of the 6th Census of Sports Site, there were 679,700 total sports venues with an area of 612 million square meters distributed in the countryside. Among them, there were 652,400 outdoor sports venues with an area of 607 million square meters, accounting for 99.18% of the total area by the end of 2013. These public sports venues are mostly provided by government for free use, and the village committee is mainly responsible for the daily management of them [29,32]. However, in most cases, after the construction of the public sports facilities, there is no continuous funding for long-term maintenance and management. As a result, a large number of sports facilities were left idle, damaged, or occupied for other use. Apart from this, the statistics show that although the number of the public sports facilities has been increasing in last decade, the number of residents who took regular exercise (According to the definition provided by [33], those who took part in physical exercise three times or more per week, each time lasting for 30 min or more, and at a moderate intensity or above are counted as participating in regular exercise.) made up only 10.4% of rural population by the end of 2014 [33], indicating that the sports participation rate is still low. Given the positive health and social impacts of sport participation, how to increase sport participation in rural communities and what are the main influencing factors of decision-making on participating in physical exercise need to be clarified. Nevertheless, quantitative studies focusing on community sports provision in rural China are still rare. To fill this gap in the existing literature, based on the survey data from rural China this study aims to provide evidence on how the provision of community sports influences household health status through changing household decisions on the usage of public sport facilities and the participation of organized sport activities. The basic hypothesis for this study is that community sports provision increases sports participation of rural community members so as to increase their health status.

## 3. Data and Descriptive Analysis

### 3.1. Data

The data used in this study were collected from a rural household survey of eight provinces (i.e., Liaoning, Hebei, Zhejiang, Guangdong, Sichuan, Hubei, Shaanxi, and Jiangxi) conducted by the China Center for Agricultural Policy (CCAP) of Peking University in December 2018. Considering the representativeness of the sample, a random sampling method was used: counties were randomly selected from the surveyed provinces. In each county, three townships were randomly selected. Similarly, a maximum of two villages were randomly selected from each township. In each village, 10–15 households were randomly selected to take the questionnaire interview. In total, the dataset includes 3468 rural households from 326 villages in 165 townships in 55 counties in the eight provinces in China.

During the face-to-face interview, households were asked about information on their participation in public sports activities in 2018. Specifically, this study focuses on their use of public sports facilities and their participation in organized sports competitions. Therefore, there were related questions, including (1) whether they participated in community sports (either using public sports facilities in the village or taking part in sports activities organized by the village) in 2018; (2) whether the public sports facilities were still in use in 2018; (3) whether any public sports activities were organized in their village. The surveyed households were also asked to report their health status, including (1) self-assessment of the health status of each household member; (2) whether their members were diagnosed with having diabetes; (3) whether their members were diagnosed with having high blood pressure (As diabetes and high blood pressure are the most common chronic diseases in rural China [34], these incidences were selected as variables measuring public health in this study); (4) whether their members got ill in 2018; (5) whether their members stayed in hospital for a disease in 2018. Additionally, the questionnaire interviews covered the basic information on household characteristics, such as household size, income level, and demographic structure, etc.

Moreover, to obtain the information on provision of public sports services in the surveyed villages, one-to-one, face-to-face questionnaire interviews with village leaders who were responsible for community sports management were also conducted. The village leaders were enquired about (1) whether their villages had public sports facilities; (2) whether their villages organized public sports activities in 2018. The descriptive statistics of some key variables are presented in Table 1 and Table 2.

### 3.2. Community Sports Provision and Social Inclusion

Community sports provision, for the purpose of this study, is defined as provision of public sports facilities (such as basketball courts, table tennis tables, outdoor fitness paths, or multi-purpose sports facilities) and organization of public sports activities. Table 1 shows that 264 of the surveyed villages provided community sports in 2018, accounting for 84.05% of the total sample. Among them, 262 villages (80.37%) had public sports facilities while 166 villages (50.92%) organized public sports activities. In contrast, about 15.95% (52) of the sample village leaders stated that their villages had neither public sports facilities nor organized sports activities. In other words, households from those villages still had no access to community sports provision.

According to Duhaime et al. [35], social inclusion is a measure of access to and participation in various networks of emotional, social, and material support. In the field of sport research, Bailey [36] pointed out if sport is to be involved in the process of social inclusion, it is essential that people have opportunities to participate. Thus, in this study the percent of households participating in community sports is adopted as a proxy of the degree of social inclusion. As it can be seen in Table 1, 39.39% (1366) of the sampled households participated in rural public sports services in 2018. To be specific, 35.84% (1243) of the surveyed households used the public sports facilities in their own villages while only 12.66% (439) of them participated in public sports activities organized there. These figures indicate that the degree of social inclusion of rural public sports services in the study areas was still relatively low.

Meanwhile, about 62.77% (2177) of the sample households reported that their villages had public sports facilities. The percentage of these facilities still in use was approximately 95.91% (2088). About 28.78% (998) of the sample households reported that their villages organized public sports activities in 2018. Comparing the data on public sports services collected from households with those collected from village leaders, it can be found that 22.03% (764) of the sample households did not know that their villages have public sports facilities, while 33.42% (1159) of them did not know about the public sports activities organized by their villages. This is possibly due to lack of information publicity. Some households still had no access to the information about related policies and events.

### 3.3. Community Sports Provision and Public Health

As suggested by Schmitt and Schmitt [27], increases in elderly population and chronic diseases will place a strain on public health in Asian developing countries in the 21st century. Measures aiming at assuring the provision of health-related services and preventing the chronic diseases can be regarded as effective enhancement of public health [37]. Considering data availability, indicators (i.e., whether or not having family member(s) diagnosed with diabetes and high blood pressure; whether or not having ill family member(s) in 2018; and whether or not the sick member(s) stayed in hospital) are used to measure the health status of the sampled households. Table 2 shows that in total, 319 (9.2%) households had members with diagnosed diabetes, while 1287 (37.11%) households had members being diagnosed with high blood pressure. Of the surveyed households, 1470 (42.39%) stated that they had member(s) who were ill in 2018. Among them, 769 (22.17%) households reported that their sick members had experienced a hospital stay.

Moreover, in order to preliminarily investigate the effects of public sports services on household health, each of the four indicators were divided into two groups in terms of whether the household participated in community sports. By comparing the figures of each indicator between the two groups, it can be found that the number of the households who were non-participators in community sports was about twice as much that of participating households. To some extent, this indicates that participating in public sports services could decrease the probability of getting ill, especially with some chronic diseases such as diabetes and high blood pressure or staying in hospital for serious diseases.

## 4. Empirical Strategy

Based on the above discussion, two Probit models for examining the effects of community sports provision on social inclusion and public health are specified as follow:

(1) $$Participation_{i}=\alpha_{0}+\sum^{\text{​}}\beta_{1j}PSS_{ij}+\beta_{2}HH_{i}+\beta_{3}Z_{i}+\varepsilon_{i}$$

(2) $$Illness_{i}=\alpha_{1}+\sum^{\text{​}}\beta_{4j}PSS_{ij}+\beta_{5}Z_{i}+\mu_{i}$$

where $\varepsilon_{i}$ and $\mu_{i}$ are random disturbance terms assumed to be independent and identically distributed with standard cumulative distribution.

In these models, two bivariate variables, “Participation in community sports” ($Participation_{i}$) and “Having ill members” ($Illness_{i}$), were used as dependent variables measuring the degree of social inclusion of the public sports services and health status for household i, respectively. $Participation_{i}$ equals to one if household i participated in community sports in 2018 and zero otherwise, whilst $Illness_{i}$ equals to one if household i had ill member(s) in 2018 and zero otherwise.

With respect to the explanatory variables on the right-hand side of the equations, the variable of interest here is $PSS_{ij}$ (*j* = 2), which denotes provision of community sports in the village where household i located. It consists of two dummy variables, namely “Having public sports facilities” that equals to one if the village had public sports facilities and zero otherwise, and “Having organized sports activities” that equals to one if the village organized sports activities and zero otherwise. The coefficients $\beta_{1j}$ and $\beta_{4j}$ demonstrate the impacts of public sports services on the degree of social inclusion and the status of public health. It is conjectured that $\beta_{1j}$ are positive and $\beta_{4j}$ are negative, as providing sports is expected to increase the probability of household participation, which in turn decreases the probability of getting ill for household members.

Moreover, $Z_{i}$ is a vector of household characteristics that may affecting household i’s decision regarding participation in community sports, as well as household heath status, including household size, average age of household members, fraction of children, fraction of elderly people, fraction of female, fraction of members with high school education or above, and income level. Among them, household size is defined as the number of the household members who lived in their own villages for more than nine months a year. It is selected as a proxy for actual demand for public sports services. Households of larger size could need more opportunities to get every member involved in public physical exercises. This also indicates that they have greater likelihood to have sick member(s). The demographic characteristics (such as average age of household members, fractions of children, elderly people, female, and members with high school education or above, and income per household member (As the income from off-farm work takes the largest share in total household income, the average income level of household members could be regarded as an exogenous variable in both probit models.) are used to measure the socioeconomic status of the household. They also reflect the effects of the gender, age, and education structure on household participation decisions on pubic sports activities and health status. Particularly for a household, the higher the average age of the members and the fraction of elderly people are, the less likely that household is to participate in community sports and the more likely it is to have member(s) getting ill. The income level reflects the economic status of the household and is expected to have positive impacts on both household participation in community sports and household health [38]. It is hypothesized that as the income level increases, the probability of participation increases and that of having sick members decreases accordingly.

In addition, it is assumed that the household head is the decision maker of participation in community sports. Therefore, household head characteristics ($HH_{i}$) such as age, gender, educational level, and health status can be considered as important factors affecting the household’s participation decisions and are included in Equation (1). Concretely, it is conjectured that the higher the decision-maker’s education level is, the higher the probability of participating in sports will be [38]. The variable “health status” is derived based on the decision-makers’ self-assessment on their own health states. It is treated as a pseudo-categorical variable in this study. This is to say, healthy = 1, relatively healthy = 2, generally healthy = 3, unhealthy = 4, very unhealthy = 5. The health status of the decision maker is expected to have positive influence on participating in community sports. The variables used in later analysis are listed in Table 3.

Separately estimating the two probit regressions may be biased. The plausible reason could be that the two dependent variables are jointly determined by some of the same independent variables. Thus, the single-equation estimation may suffer from simultaneity bias due to the correlation between the disturbance of each equation and the dependent variables. To obtain more efficient estimates, the seemingly unrelated bivariate probit (SUBP) model is applied to systematically estimate these two equations [39,40]. Moreover, in practice, since the provision of community sports influences household health mainly through encouraging the household to participate in public sports activities, another endogeneity problem may be cause by the correlated error terms among the two equations. As suggested by Thuo et al. [41] and Filippini et al. [42], the recursive bivariate probit (RBP) model is employed to control the potential endogenous variable ($Participation_{i}$) in the system.

## 5. Estimation Results

The estimation results of both the seemingly unrelated bivariate probit (SUBP) model and the recursive bivariate probit (RBP) model are presented in Table 4. Firstly, considering the results of Wald tests, the value of ρ = −0.048 for the SUBP model is not statistically significant. Thus, the null hypothesis that *ρ* = 0 can be accepted, indicating that the probability that a household participated in community sports was not related to the probability of having sick member(s) through unobserved effects captured in the model’s error terms. In the RBP model, the value of ρ = 0.907 is positive, revealing that participation in community sports and having ill member(s) are complementary variables. They move in the same direction. This is to say that for a household having member(s) getting ill was related to the decision to participate in public sports services. The statistically significant result of the Wald test for *ρ* = 0 indicates that the null hypothesis that participation in community sports ($Participation_{i}$) is exogenous can be rejected, suggesting that the decision to participate in community sports and whether or not having sick member(s) were jointly determined. Therefore, the RBP model outperforms the SUBP model and the remaining analysis is focused on the results of the RBP model. The corresponding marginal effects of the statistically significant variables in the RBP model are calculated as sample means and given in Table 5.

As shown in Table 4, the estimated coefficients of most variables have the expected signs. Specifically, the provision of public sports facilities has a positive and significant effect on household participation in community sports, while having a negative and significant effect on the probability that a household had sick member(s). This indicates that providing public sports facilities in the village increases the likelihood that a household will use them to do physical exercises and simultaneously decreases the probability for members getting ill. The marginal effects of this variable provide more information about these effects. According to the calculated values of the marginal effects (see Table 5), the households who lived in the village with public sports facilities are about 9.70% more likely to participate in public sports, while being about 6.50% less likely to have sick member(s). Another indicator of community sports provision, organization of public sports activities, has significant and positive effect on participating in community sports, but no statistically significant effect on public health. This is partly in line with the previous expectations in Section 4, suggesting that although organizing public sports activities arouses the enthusiasm of households for getting involved in physical exercises, the current frequency and quality of the organized sports activities still cannot meet the demand of enhancing public health in rural areas.

Besides, consistent results of the two regressions (Equations (1) and (2)) are obtained for the parameters associated with age and gender structure of household and income level. The fractions of female and children in household members are found to have negative effects on the household’s decision to participate in public sports and its health status, while the fraction of elderly people has a positive effect. The average income of each household member is negative and statistically significant in both of the two regressions, suggesting that raising the household income level decreases the probability of participating in community sports, as well as the probability of having member(s) getting ill. Concretely, a 10% increase in income per household member will reduce the probability of participation and of having sick member(s) by about 0.12% and 0.42%, respectively.

The findings on participation in community sports (Equation (1)) also highlight the role of household characteristics in related decision making. In particular, the educational level of the household head has significant and positive effects on household participation in community sports. The well-educated household head increases the probability for the household to use the public sports facilities or to take part in the public sports activities organized in the village.

## 6. Conclusions

This paper examined the effectiveness of community sports provision on social inclusion and public health using the micro-level data from a household survey conducted in eight provinces of rural China. The empirical results show that the provision of community sports was partially effective in promoting inclusion and health in rural areas. Specifically, constructing public sports facilities is effective in increasing participation in public sports and decreasing the risk of getting ill. In contrast, organizing public sports activities is effective in increasing the chance of playing sports. Nonetheless, it is ineffective in promoting health. It also should be noted that economic growth (reflected in the increase in income level) may enhance public health through advancing medical technologies and improving sanitary conditions instead of encouraging participation in sports.

Based on the results of this study, the following recommendations on future policy design for provision of public sports services in rural China are carefully raised. First, constructing public sports facilities in every village should be done to ensure that all households have access to them. In the short term, this can increase the opportunity for households to participate in public sports activities, while in the long term, this can improve the household’s health status through facilitating the formation of a physically active lifestyle.

Second, improving the quality of community sports provisions and managing them effectively so as to ensure households make full use of them. On one hand, many public sports facilities fell out of use due to poor maintenance. Therefore, more funds and a labor force need to be invested in to change this situation. On the other hand, more attention should be paid to increase the frequency and fun of the public sports activities to attract more households.

Third, providing access to the information and instruction on public sports should be a priority. This can also help increase the participation in public sports activities, as some households reported that they knew nothing about the public sports facilities or they were not informed of the organized sports events in the village. Moreover, many rural households were unfamiliar with some new sports facilities, making them question how to use them. Households may also have little knowledge on the health benefits of engaging in physical exercise. Delivering related instruction not only provides training to households, but also reveals the potential health effects of physical activities.

## Figures and Tables

**Table 1:** Statistics of the variables related to community sports provision in the sample.

Levels	Categories	Number	Proportion (%)
Households	Participated in public sports services in 2018	1366	39.39
	Used public sports facilities	1243	35.84
	Participated in public sports activities	439	12.66
	Knowing that their own villages had public sports facilities	2177	62.77
	Knowing that their own villages organized public sports activities	998	28.78
	Had public sports facilities that were still in use in their own village in 2018	2088	60.21
	Total number	3468	-
Villages	Provision of public sports services in 2018	264	84.05
	Had public sports facilities	262	80.37
	Organized public sports activities	166	50.92
	None provision of public sports services in 2018	52	15.95
	Total number	326	-

Data source: Household survey in eight provinces conducted by CCAP (China Center for Agricultural Policy).

**Table 2:** Descriptive statistics of the variables related to public health in the sample.

Level	Categories	Number	Proportion (%)
Households	Had members diagnosed with diabetes	319	9.20
	Among them		
	Participator of public sports services	118	36.99
	Non-participator of public sports services	201	63.01
	Had members diagnosed with high blood pressure	1287	37.11
	Among them		
	Participator of public sports services	514	39.94
	Non-participator of public sports services	773	60.06
	Had ill members in 2018	1470	42.39
	Among them		
	Participator of public sports services	546	37.14
	Non-participator of public sports services	924	62.86
	Had members staying in hospital in 2018	769	22.17
	Among them		
	Participator of public sports services	288	37.45
	Non-participator of public sports services	481	62.55
	Total number	3468	-

Data source: Household survey in eight provinces conducted by CCAP.

**Table 3:** Description of the variables used for model estimation.

Variables	Obs.	Mean	Std. dev.	Min	Max
Participation in public sports services (1 = yes; 0 = no)	3468	0.394	0.489	0	1
Having members getting ill (1 = yes; 0 = no)	3468	0.424	0.494	0	1
Having public sports facilities in the village where the household located (1 = yes; 0 = no)	3468	0.796	0.403	0	1
Having organized sports activities in the village where the household located (1 = yes; 0 = no)	3468	0.502	0.500	0	1
Gender of household head (1 = male; 0 = female)	3468	0.927	0.261	0	1
Age of household head (Years)	3468	57.893	10.405	12	92
Educational years of household head (Years)	3468	6.897	3.251	0	16
Health status of household head	3468	2.299	1.087	1	5
Household size (People)	3468	2.866	1.500	1	12
Average age of household members (Years)	3468	50.10	14.41	2	86
Fraction of children in household members	3468	0.116	0.181	0	1
Fraction of elderly people in household members	3468	0.219	0.347	0	1
Fraction of female in household members	3468	0.507	0.212	0	1
Fraction of household members attended high school education or above	3468	0.274	0.287	0	1
Income per household member (CNY)	3468	14,206	10,073.88	250	125,200

Data source: Household survey in eight provinces conducted by CCAP.

**Table 4:** Effects of community sports provision on social inclusion and public health.

Variables	(1) Seemingly Unrelated Bivariate Probit (SUBP) Model		(2) Recursive Bivariate Probit (RBP) Model	
	Participation	Illness	Participation	Illness
Provision of public sports facilities (yes = 1; no = 0)	0.435 *** (0.084)	−0.183 *** (0.056)	0.363 *** (0.078)	−0.194 *** (0.054)
Organization of public sports activities (yes = 0; no = 0)	0.131 ** (0.057)	0.088 (0.045)	0.110 ** (0.054)	0.092 (0.042)
Household size	0.004 (0.028)	0.113 *** (0.021)	−0.001 (0.026)	0.092 *** (0.019)
Gender of household head (male = 1; female = 0)	−0.198 * (0.106)		−0.127 (0.086)	
Age of household head (Years)	0.002 (0.004)		0.005 * (0.003)	
Educational level of household head (Years)	0.020 ** (0.010)		0.016 ** (0.008)	
Heath status of household head	−0.101 *** (0.029)		−0.227 *** (0.023)	
Average age of household members (Years)	−0.011 ** (0.005)	0.013 *** (0.004)	−0.008 (0.005)	0.007 * (0.003)
Fraction of children (≤14)	−0.613 ** (0.297)	−0.222 (0.221)	−0.519 * (0.287)	−0.054 (0.209)
Fraction of elderly people (≥65)	0.040 (0.122)	0.178 * (0.093)	0.019 (0.115)	0.164 * (0.088)
Fraction of female	−0.126 (0.139)	−0.112 (0.105)	−0.129 (0.132)	−0.162 * (0.098)
Fraction of members with high school education or above	−0.290 ** (0.127)	0.213 ** (0.092)	−0.161 (0.116)	0.084 (0.087)
Income per household member (CNY) ^a^	−0.058 (0.039)	−0.108 *** (0.031)	−0.101 *** (0.037)	−0.103 *** (0.029)
Participation in public sports services (yes = 1; no = 0)				−1.622 *** (0.059)
Constant	−0.191 (0.490)	−0.123 (0.037)	−0.196 (0.477)	0.367(0.352)
Rho	−0.048		0.907 ***	
Log pseudolikelihood	−3542.814		−3501.923	
Wald chi2	200.41 ***		1214.79 ***	
Wald test of rho = 0	1.713		77.849 ***	
Number of observations	3468		3468	

This table shows the effects of community sports provision on household participation in public sports and health, and the results are obtained by estimating the system (1) with Stata 15.0. Significance level: *** *p* < 0.01; ** *p* < 0.05; and * *p* < 0.1. The values in parentheses are robust standard errors. The superscript “a” means that variables are in logarithms for elimination of heteroscedasticity.

**Table 5:** Effects of statistically significant variables in recursive bivariate probit (RBP) model.

Variables	Participation	Illness
	Pr. = 0.395	Pr. = 0.425
Provision of public sports facilities	0.097 ***	−0.065 ***
Organization of public sports activities	0.026 **	
Household size		0.043 ***
Age of household head	0.001 *	
Educational level of household head	0.004 **	
Health status of household head	−0.022 ***	
Average age of household members		0.005 ***
Fraction of Children	−0.124 *	
Fraction of elderly people		0.069 *
Fraction of female		−0.045 *
Income per household member	−0.012 ***	−0.042 ***
Participation in public sports services (yes = 1; no = 0)		−0.060 ***

Significance level: *** *p* < 0.01; ** *p* < 0.05; and * *p* < 0.1. The values in parentheses are robust standard errors. Pr. is the predicted probability.
